# Reassortants of the Highly Pathogenic Influenza Virus A/H5N1 Causing Mass Swan Mortality in Kazakhstan from 2023 to 2024

**DOI:** 10.3390/ani14223211

**Published:** 2024-11-08

**Authors:** Kulyaisan T. Sultankulova, Takhmina U. Argimbayeva, Nurdos A. Aubakir, Arailym Bopi, Zamira D. Omarova, Aibarys M. Melisbek, Kobey Karamendin, Aidyn Kydyrmanov, Olga V. Chervyakova, Aslan A. Kerimbayev, Yerbol D. Burashev, Yermukhanmet T. Kasymbekov, Mukhit B. Orynbayev

**Affiliations:** 1 Research Institute for Biological Safety Problems (RIBSP), Gvardeiskiy 080409, Kazakhstan; sultankul70@mail.ru (K.T.S.); 98.constantine.98@gmail.com (T.U.A.); nurdos.aubakirov@mail.ru (N.A.A.); arailim__b@mail.ru (A.B.); zarina-omarova-80@mail.ru (Z.D.O.); aibarysmelisbek@gmail.com (A.M.M.); ovch@mail.ru (O.V.C.); aslan_kerim@mail.ru (A.A.K.); yerbol.bur@gmail.com (Y.D.B.); 2 Research and Production Center of Microbiology and Virology, Almaty 050010, Kazakhstan; kobey.karamendin@gmail.com (K.K.); kydyrmanov@yandex.kz (A.K.); kasymbek.ermuxan@mail.ru (Y.T.K.)

**Keywords:** Caspian Sea, avian influenza, wild birds, mute swan, whooper swan, PCR, phylogenetic analysis

## Abstract

In the winter of 2023/2024, the mass death of swans was observed on Lake Karakol on the eastern coast of the Caspian Sea. From 21 December 2023 to 25 January 2024, 1132 swan corpses (*Cygnus olor*, *Cygnus cygnus*) were collected and disposed of on the coast by veterinary services and ecologists. Biological samples were collected from 18 birds for analysis at different dates of the epizootic. It was found that the influenza outbreak was associated with a high concentration of migrating birds at Lake Karakol as a result of a sharp cold snap in the northern regions. At different dates of the epizootic, three avian influenza A/H5N1 viruses of clade 2.3.4.4.b were isolated from dead birds and identified as highly pathogenic viruses (HPAIs) based on the amino acid sequence of the hemagglutinin multi-base proteolytic cleavage site (PLREKRRRKR/G). A phylogenetic analysis showed that the viruses isolated from the swans had reassortations in the PB2, PB1, and NP genes between highly pathogenic (HP) and low-pathogenic (LP) avian influenza viruses. Avian influenza viruses A/*Cygnus cygnus*/Karakol lake/01/2024(H5N1) and A/*Mute swan*/Karakol lake/02/2024(H5N1) isolated on 10 January 2024 received PB2, PB1, and NP from LPAIV, while A/*Mute swan*/Mangystau/9809/2023(H5N1) isolated on 26 December 2023 received PB1 and NP from LPAIV, indicating that the H5N1 viruses in this study are new reassortants. All viruses showed amino acid substitutions in the PB2, PB1, NP, and NS1 segments, which are critical for enhanced virulence or adaptation in mammals. An analysis of the genomes of the isolated viruses showed that bird deaths during different periods of the epizootic were caused by different reassortant viruses. Kazakhstan is located at the crossroads of several migratory routes of migratory birds, and the possible participation of wild birds in the introduction of various pathogens into the regions of Kazakhstan requires further study.

## Simple Summary

The results of this study allowed us to identify and characterize the influenza virus that caused mass mortality in swans in the winter of 2023/2024 at Lake Karakol (the eastern coast of the Caspian Sea). The findings highlight the need for the continued surveillance of major migration routes in this region. The monitoring of avian influenza viruses and the genome sequencing of the identified viruses may play an important role in improving our understanding of the intercontinental transmission of influenza viruses and the early detection of newly emerging reassortant strains.

## 1. Introduction

Avian influenza virus (AIV) is one of the most well-known pathogens, and its constant circulation has a significant impact on public health and the global economy. AIVs are segmented, single-stranded RNA viruses of the *Orthomyxoviridae* family and are highly variable [1]. The genetic and antigenic variability of surface glycoproteins HA and NA is most pronounced. Up to now, 16 major antigenic variants of HA and nine antigenic variants of NA in birds have been identified [2]. In addition to classification based on the antigenic properties of HA and NA, HPAIs are categorized into two groups based on their pathogenicity to avian species: highly pathogenic and low-pathogenic (HPAIV and LPAIV). The vast majority of avian influenza viruses are low-pathogenic (LPAI) and cause either a mild course of disease or no symptoms, depending on the virus and the infected species. However, two influenza subtypes, H5 and H7, can also occur as highly pathogenic viruses (HPAIs).

The beginning of the largest avian influenza epizootic in modern history, which is still ongoing, was the outbreak of the highly pathogenic avian influenza virus H5N1 in 1996 in Guangdong Province of China [3,4]. In 2005, the H5N1 virus belonging to clade 2.2 caused the first major outbreak among wild birds in Qinghai Lake in China, and then, with the migration of wild birds, the virus spread from Asia through Siberia to Europe, the Middle East, and Africa in a few months [5,6]. In the same year, the first cases of avian influenza with mass mortality of poultry were registered in seven regions of Kazakhstan. Highly pathogenic avian influenza A(H5N1) has spread widely among wild and domestic birds in Asia, Europe, and Africa. By the end of 2021, it was detected in North America, indicating further intercontinental spread [7]. Outbreaks of highly pathogenic avian influenza H5N1 in poultry have caused enormous economic losses to the poultry industry, and its sporadic transmission to humans in a number of countries has emphasized its potential public health threat. Since 2003, a total of 889 sporadic human cases of A(H5N1) caused by different clades of the HPAI A(H5N1) virus have been reported in 23 countries [8]. The number of poultry affected by these outbreaks is unknown, but probably around hundreds of millions [6].

The mortality of wild waterfowl from infection with highly pathogenic avian influenza viruses (HPAIVs) is a rare event. In wild birds, HPAI A/H5N1 was first reported in 2002 in Hong Kong [9]. Wild birds are the natural reservoir of influenza A viruses that usually cause asymptomatic infection. However, since 2005, there have been numerous outbreaks caused by A/H5N1 HPV, with the mass deaths of wild birds in Asia [10,11], Europe [12,13,14], Africa [6], and North America [15]. These events have raised concerns that migratory birds may play a role in the transmission of HPAIV, and there is a need for ongoing studies of this pathogen in wild bird populations.

Several migration routes pass through the territory of Kazakhstan, which can facilitate the spread of the influenza virus from the flyways of Southeast Asia to the European and North African flyways and vice versa [16]. The Caspian Sea and the Caspian water bodies are important areas for the seasonal migrations of many migratory birds of Eurasia. Wild birds use this region for wintering and nesting, and for some birds, this region is a transit region on their way to their further migration to the places of North African and Indo-Pakistani wintering grounds [17].

Since 2006, several cases of disease and mass mortality among wild birds caused by HPAIV have been recorded in the northern Caspian Sea region. The first outbreak of HPAIV, with a mass mortality of at least 400 whooper swans, occurred in 2005 [18], and the most recent outbreak occurred in 2022, when more than 30,000 gulls (*Ichthyaetus melanocephalus*, *Larus cachinnans*), terns (*Hydroprogne caspia*), and pelicans (*Pelecanus crispus*) died [16]. Single deaths of wild birds on the Caspian Sea coast are registered almost annually, but due to the lack of a unified program for monitoring wildlife diseases, many bird deaths often go unnoticed. Interest in this problem arose in late 2023 and early 2024 when the mass mortality of swans began on the eastern shore of Kazakhstan’s Caspian Sea water area in the Mangystau region.

Given the constant circulation of the influenza virus in this region in birds and the constantly changing nature of these viruses, in this study, we aimed to investigate the genetic evolution, reassortment, and virulence of H5 viruses detected in dead swans from 2023 to 2024.

## 2. Materials and Methods

### 2.1. Ethics Statement

All studies on birds were conducted in accordance with the guidelines of the Animal Care and Use Committee of the Research Institute for Biological Safety Problems (RIBSP), and all animal study protocols were approved by RIBSP (Permit number: 1-14-07-2023).

### 2.2. Ecological Features of the Study Area

**Figure 1:**
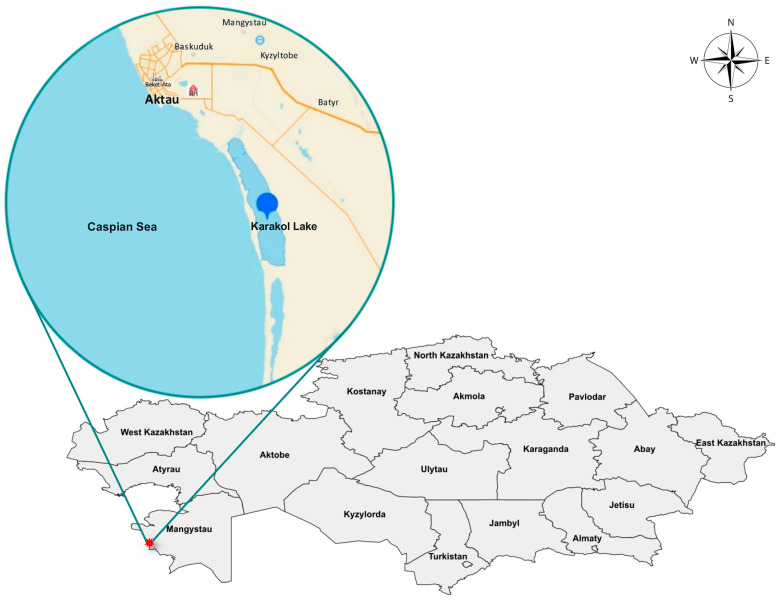
Wild bird death site in the winter of 2023/2024.

The artificial lake (reservoir) Karakol is located on the eastern shore of the Caspian Sea, separated from the sea by a narrow, low dam (Figure 1); it is a vast shallow flowing reservoir, about 10–12 km long and up to 2–4 km wide, with a total area of 4500 hectares, with numerous islands. Islets and the western shore are covered with reeds. The thicket has created favorable conditions for nesting, wintering, and rest during migration for a huge number of diverse waterfowl. Lake Karakol receives a constant supply of warm water from the Thermal Power Plant (CHP). As a result, the water here does not freeze even during the coldest winters.

Lake (reservoir) Karakol is a specially protected natural area of the Karagiye-Karakol State Zoological Reserve of national significance, created in 1986. The lake is a wintering and resting place for tens of thousands of birds. Over 80 species of migratory birds visit, including 21 species of birds listed in the Red Book of Kazakhstan and the Red Book of the Commonwealth of Independent States member states (CIS), as well as in the «Red List of Threatened Species» of the International Union for Conservation of Nature and Natural Resources (IUCN): Bewick’s swan (*Cygnus columbianus bewickii*), whooper swan (*Cygnus cygnus*), Dalmatian pelican (*Pelecanus crispus*), pink pelican (*Pelecanus onocrotalus*), squacco heron (*Ardeola ralloides*), Egyptian egret (*Bubulcus ibis*), little egret (*Egretta garzetta*), pink flamingo (*Phoenicopterus roseus*), Eurasian eagle-owl (*Bubo bubo*), Eurasian spoonbill (*Platalea leucorodia*), glossy ibis (*Plegadis falcinellus*), Pallas’s gull (*Ichthyaetus relictus*), white-tailed eagle (*Haliaeetus albicilla*), and others. Every year, many swans spend the winter in Karakol. However, this is a highly “mobile” wintering ground, as they arrive when their primary wintering sites are covered with ice and no longer suitable for their stay. At this time, a significant number of mute swans can be seen in Karakol.

### 2.3. Sampling

After the detection of the first cases of bird deaths, the veterinary service of the Ministry of Agriculture of the Republic of Kazakhstan (MoA of the RK) organized measures to find out the cause of bird deaths, as well as the localization and elimination of the disease. There is no permanent program for the monitoring of diseases of wild animals and birds in Kazakhstan, and therefore, in case of emergency, the veterinary service of the RK involves various scientific organizations. To determine the cause of the mass death of wild birds in the winter of 2023/2024, specialists from the Research Institute for Biological Safety Problems (RIBSP) and the Research and Production Center of Microbiology and Virology (IMV) were involved. These specialists are constantly involved (by the veterinary service of Kazakhstan) in cases of disease of wild animals and birds in various regions of Kazakhstan.

The sampling was carried out in the presence of state veterinarians of the Ministry of Agriculture of the Republic of Kazakhstan. The specialists of the RIBSP carried out the sampling in the presence of the chief veterinarian of the Republic of Kazakhstan and the chief veterinarian of the Mangystau region of the Republic of Kazakhstan; the specialists of the IMV carried out the sampling in the presence of veterinary specialists of the Mangystau region. Bird mortality reports throughout the epizootic period were received from state veterinary and environmental organizations. A total of 15 wild bird samples were collected by two organizations during different periods of bird mortality. On 26 December 2023, IMV employees collected 37 biological samples from eleven mute swans (*Cygnus olor*) and one common goldeneye (*Bucephala clangula*). From 9 to 10 January 2024, RIBSP specialists collected 15 samples from one adult whooper swan and two young mute swans. All samples were collected from dead birds.

Swabs from the oropharynx and cloaca, as well as tissue samples (lungs, liver, and heart) from the dead birds, were placed in numbered cryotubes with transport medium, and documentation of the collected material was carried out according to the WHO protocol [19,20]. A sterile medium for transporting tissue samples and swabs was prepared directly in the laboratory according to the recommended method [19].

All samples were frozen and transported to the laboratory in Dewar vessels with liquid nitrogen (in the case of sampling by RIBSP personnel) and in a thermal suitcase (in the case of sampling by IMV personnel).

The tubes with the swabs and tissue samples were stored in liquid nitrogen or in a low-temperature freezer at a temperature not exceeding −70 °C until analysis.

### 2.4. Virus Isolation and Characterization

Virus isolation was carried out in 11-day-old SPF embryonated chicken eggs based on standard procedures [21]. Each sample was passed three times before the final diagnosis based on hemagglutination activity.

### 2.5. RNA Extraction

RNA was extracted from the samples using the QIAamp Viral RNA kit (Qiagen, Hilden, Germany) according to the manufacturer’s instructions.

### 2.6. Real-Time RT-PCR for Primary Detection of Influenza A Viruses

A real-time RT-PCR assay targeting a highly conserved region of the M gene was performed as described in [22]. The rRT-PCR was performed according to a method described previously [22], with 2 μL of template in 20 μL master mix using a LightCycler^®^ 2.0 Instrument Real-Time PCR System (Roche Applied Science, Penzberg, Germany).

### 2.7. Determination of the Influenza Virus Subtype

The determination of the H5 hemagglutinin subtype from isolated strains was performed using RT-PCR and subtype-specific primers [16,19].

The obtained PCR product was analyzed using agarose gel (1.5%) electrophoresis, and a 100 bp DNA ladder (Invitrogen, Taastrup, Denmark) was used as a marker.

### 2.8. RT-PCR Reaction

Both conventional RT-PCR and real-time RT-PCR (rRT-PCR) were performed using a one-step RT-PCR kit (Qiagen). All the primers and probes were synthesized using H-16 DNA/RNA/LNA Synthesizer (K&A Laborgeraete, Schaafheim, Germany). The sequences of primers, probes, and references used in this study are shown in Table S1 [22,23,24,25].

### 2.9. Sequencing and Phylogenetic Analysis

Positive samples for haemagglutinin and neuraminidase of AI H5N1 (detected by RT-PCR) were subjected to nucleotide sequencing using a primer according to E. Hoffmann et al. [25] on an Applied Biosystems 3130 genetic analyser (HITACHI, Tokyo, Japan) using the Bigdye Terminator V3.1 loop sequencing kit (Applied Biosystems, Inc., Austin, TX, USA). 

The obtained nucleotide sequences were analyzed using the Sequencher v. 4.5 program (Gene Codes Corporation, Ann Arbor, MI, USA). The nucleotide sequence was aligned using the Mega 7.0 computer program complex. A set of nucleotide sequences from the international GenBank database of the National Center for Biotechnology Information (NCBI) was used to construct a phylogenetic tree and determine the genotype (Table S2). Phylogenetic analysis of the sequences was performed using the Mega 7.0 program with the following parameters: Statistical Method: maximum likelihood; Test of Phylogeny: Bootstrap method; No. Of Bootstrap Replications: 1000; Model/Method: Kimura two-parameter model.

The calculation of genetic distances was performed using the computer program Mega 7.0 with the following parameters: Analysis: Distance Estimation; Variance Estimation Method: Bootstrap method; Model/Method: P distance [26].

## 3. Results

### 3.1. Clinical and Epidemiologic Features of the Avian Influenza Outbreak at Lake Karakol in the Winter of 2023/2024

**Figure 2:**
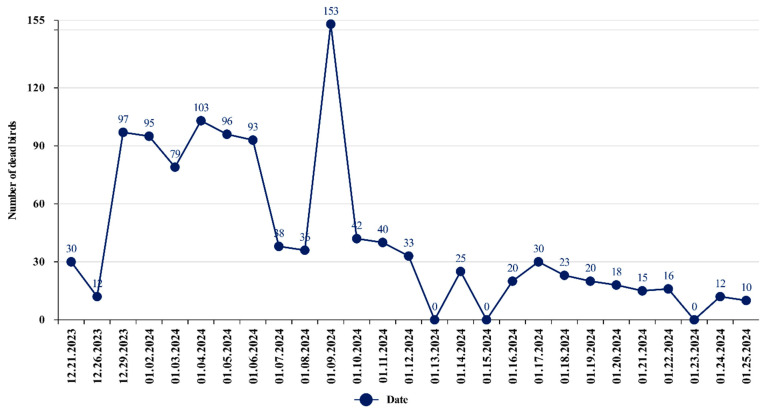
Dynamics of swan mortality on Lake Karakol in the winter of 2023/2024 (according to data from the veterinary service of the Mangystau region).

From late 2023 to early 2024, mass deaths of swans were observed on Lake Karakol in the Mangystau region. The first 30 dead swans were discovered on 21 December 2023 by inspectors of the Karagiye-Karakol State Nature Reserve in the course of protection measures. The birds continued to fall sick and die until 25 January 2024. The veterinary service of the Mangystau region collected and disposed of 1132 corpses of wild birds, including more than 90% young birds, by burning. Primarily swan carcasses were collected. Only one case of the death of a common goldeneye was noted by the employees of the Scientific and Production Center for Microbiology and Virology at the end of December. The maximum peak of bird mortality was recorded in the period from 29 December 2023 to 9 January 2024. The dynamics of swan mortality are presented in Figure 2.

**Figure 3:**
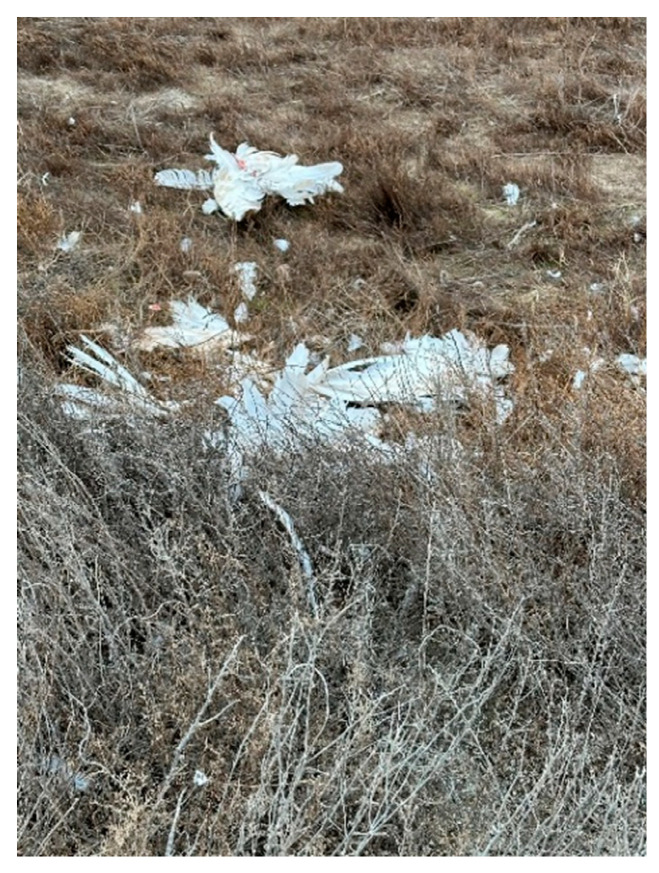
Remains of a swan’s corpse.

**Figure 4:**
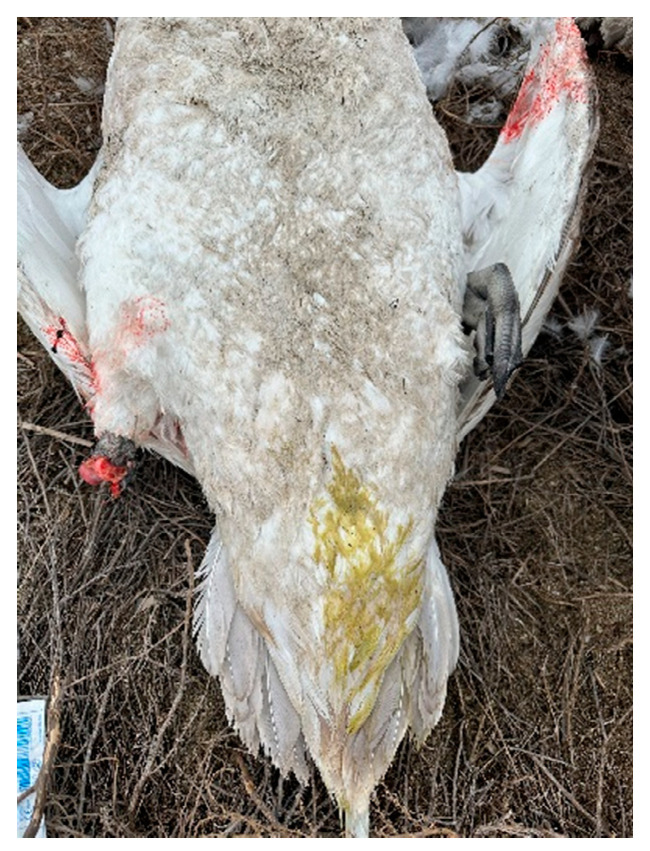
Swan corpse with signs of diarrhea and without a right leg (with a gnawed leg).

**Figure 5:**
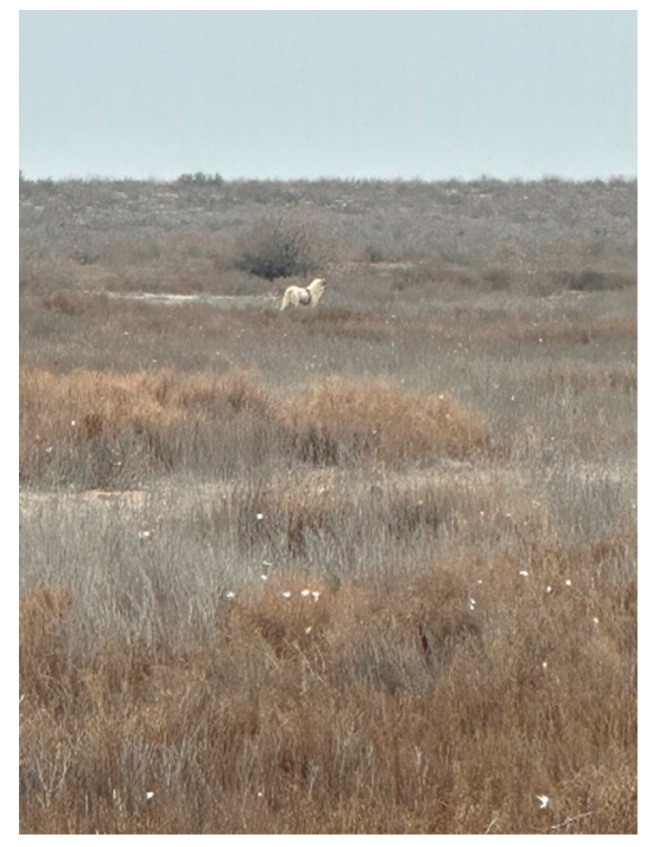
Stray dog on the shore of Lake Karakol.

It should be noted that the number of dead birds was higher. During the survey, we noted that many remains of dead and sick birds (Figure 3, Figure 4 and Figure 5) were eaten by stray dogs and wild carnivores.

**Figure 6:**
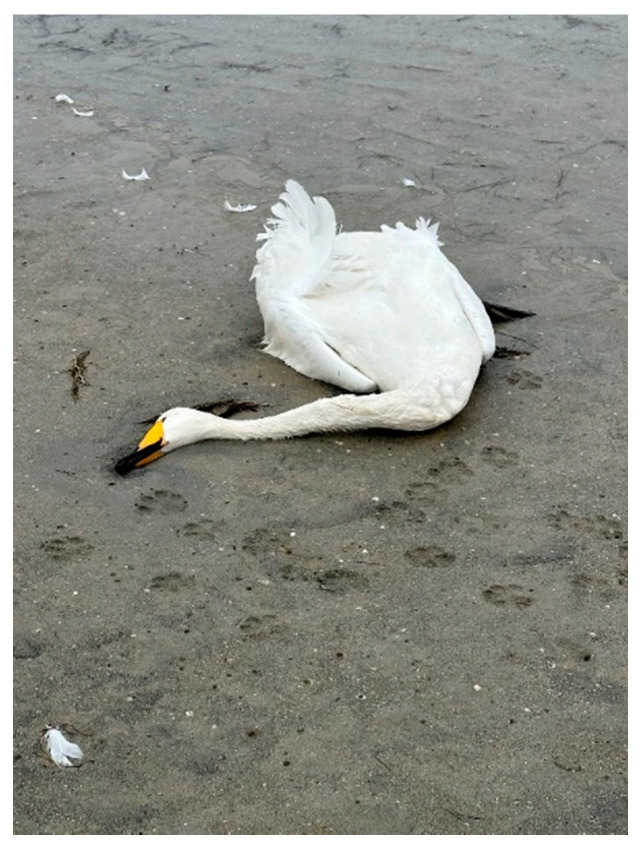
Whooper swan (*Cygnus cygnus*) (an adult).

**Figure 7:**
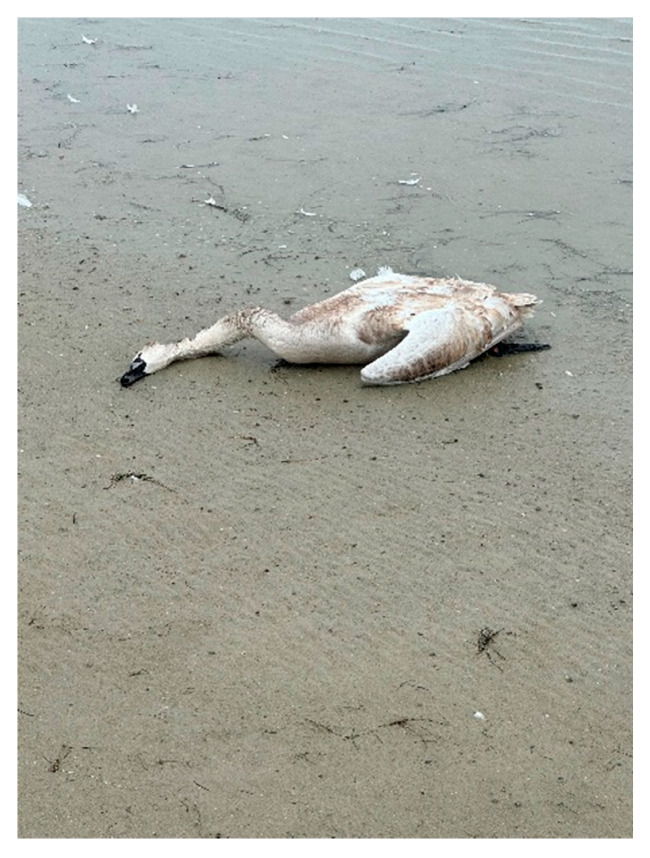
Whooper swan (*Cygnus olor*) (a cygnet).

**Figure 8:**
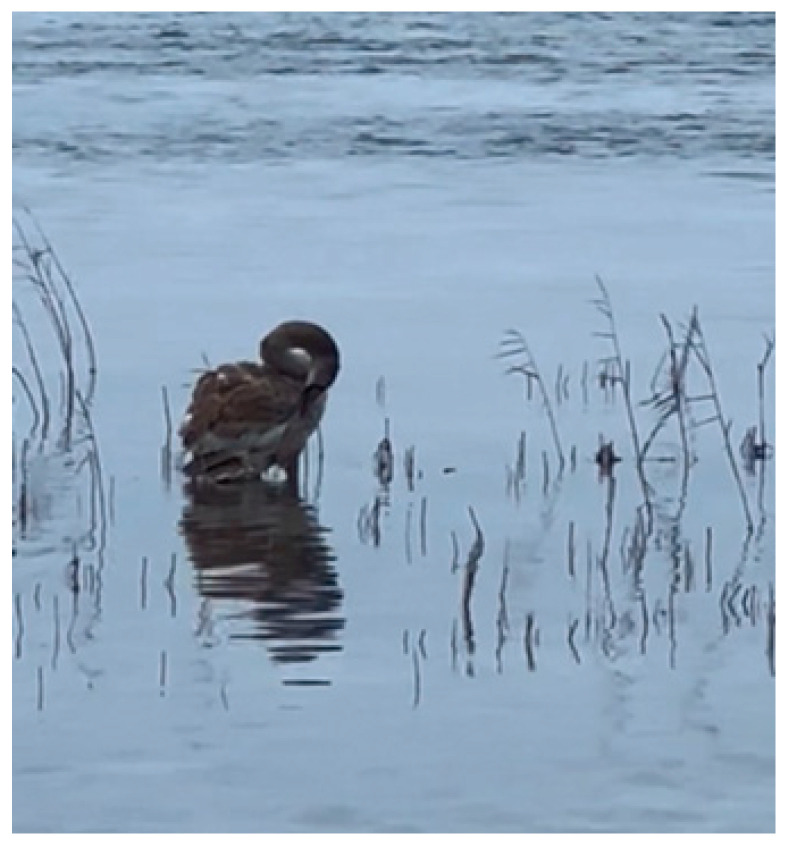
Sick bird (a cygnet).

During our survey on the shore of Lake Karakol, we found carcasses of adult and juvenile swans (Figure 6 and Figure 7), as well as two juveniles with clinical signs of disease. One of them was sitting on the water in a motionless, depressed state (Figure 8). Neurological disorders were observed in the second young swan. It was continuously circling and twirling its head (Video S1 in the Supplementary Materials). One dead bird showed signs of diarrhea (Figure 4).

**Figure 9:**
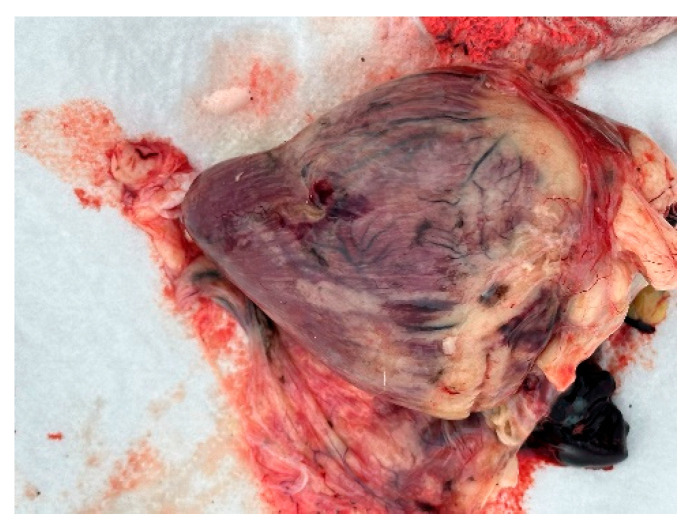
Heart. Haemorrhages in the myocard.

**Figure 10:**
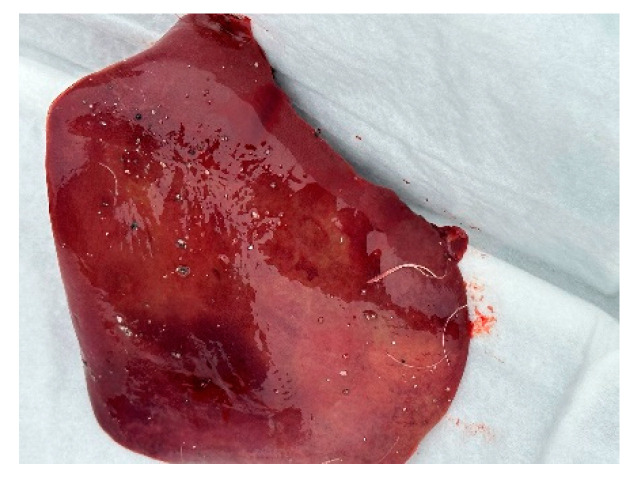
Haemorrhages in the liver.

**Figure 11:**
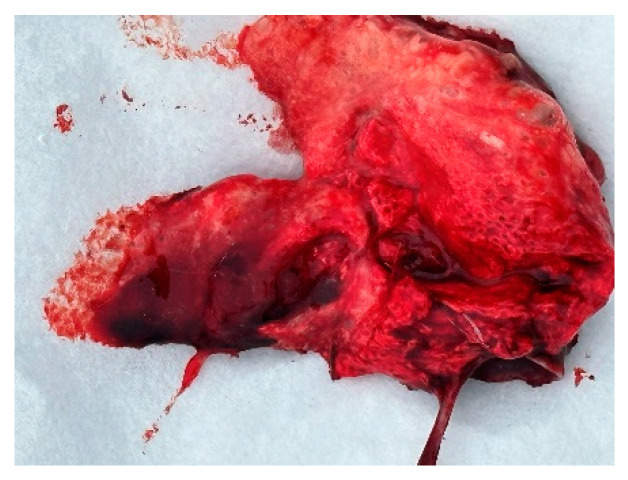
Lung edema.

Autopsies and the collection of swabs (oropharynx and cloaca) and internal organs (lungs, liver, and heart) of three swans (one adult whooper swan and two cygnets of a mute swan) were carried out in co-operation with veterinarians. The autopsy revealed hemorrhages in all internal organs (Figure 9, Figure 10 and Figure 11).

### 3.2. AIV Detection and Characterization

As a result of research conducted by two organizations (RIBSP and IMV), avian influenza virus H5N1 clade 2.3.4.4.b was isolated and identified as an HPAI virus by using the amino acid sequence of the hemagglutinin multi-base proteolytic cleavage site (PLREKRRRKR/G).

Three viruses were isolated and sequenced by Sanger. The full sequences of two viruses (A/*Cygnus cygnus*/Karakol lake/01/2024(H5N1) and A/*Mute Swan*/Karakol lake/02/2024(H5N1)) were obtained from three dead birds studied by RIBSP employees. One complete sequence of the avian influenza virus A/*Mute swan*/Mangystau/9809/2023(H5N1) was obtained via IMV (Table 1). All of the complete sequences were deposited in the international GenBank database.

In this study, we conducted a study of the genome of two isolated viruses.

To obtain a complete picture of the genetic diversity of viruses circulating during the death of the swans on Lake Karakol in the winter of 2023/2024, we used sequences of avian influenza virus A/*mute swan*/Mangystau/1-S24R-2/2024(H5N1) obtained by employees of the National Veterinary Reference Center (NVRC) and Kazakh National Research Agrarian University (KazNARU) (access numbers: PP267962 PB2, PP267963 PB1, PP267964 PA, PP267965 HA, PP267966 NP, PP267967 NA, PP267968 M, and PP267969 NS). During the epizootic period, NVRC detected influenza A/H5 virus in the dead swans via PCR. Later, the material from NVRC was transferred to KazNARU, where the complete sequence of avian influenza A/H5N1 virus was obtained [27].

**Figure 12:**
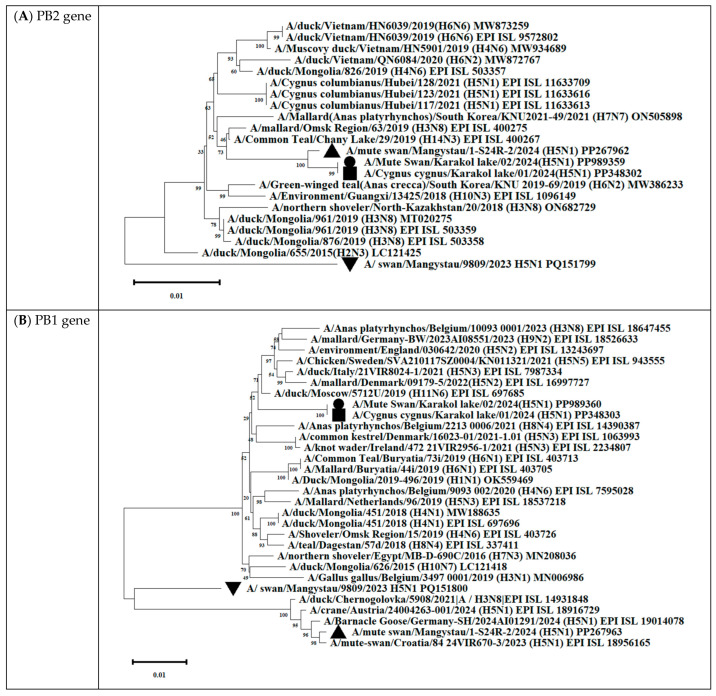

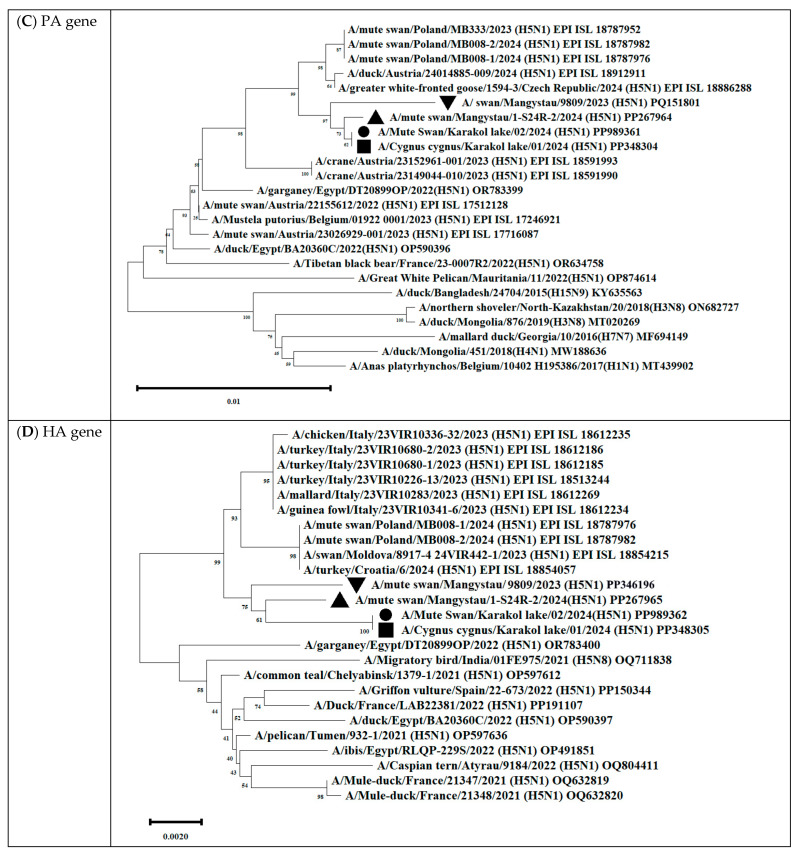

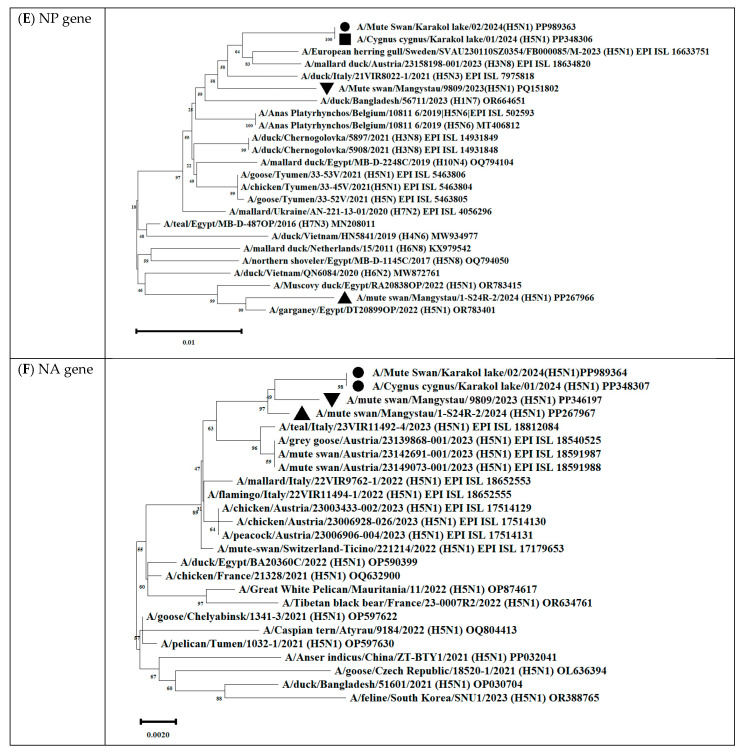

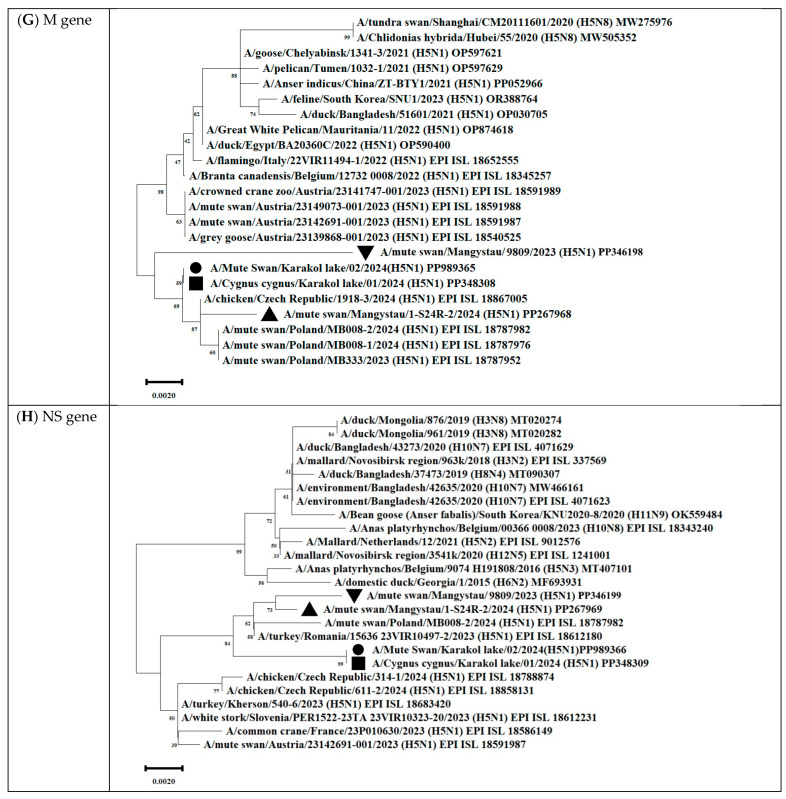
Phylogenetic trees, including complete PB2 (**A**), PB1 (**B**), PA (**C**), HA (**D**), NP (**E**), NA (**F**), M (**G**), and NS (**H**) genes, of Kazakhstani HPAIV H5N1 strains isolated from swans on the coast of Lake Karakol, located on the eastern shore of the Kazakhstani part of the Caspian Sea from 2023 to 2024 and publicly available sequences (GenBank). The strains investigated in this study are marked with triangles, squares, and circles: 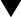
—A/*Mute swan*/Mangystau/9809/2023(H5N1); 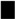
—A/*Cygnus cygnus*/Karakol lake/01/2024(H5N1); 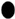
—A/Mute swan/Karakol lake/02/2024(H5N1); 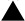
—A/mute swan/Mangystau/1-S24R-2/2024(H5N1) (virus isolated at NVRC and KazNARU by Tabynov K et al. in 2024 [27]).

Phylogenetic analysis showed that the PA/HA/NA/M/NS genes of all isolated viruses clustered with other H5Nx viruses circulating in Eurasia from 2021 to 2024 (Figure 12 and Table S3).

The analysis was based on the full-length coding sequences of the PB2, PB1, PA, HA, NP, NA, M, and NS genes. Evolutionary history was inferred using the maximum likelihood method based on Kimura’s two-parameter model implemented in Mega 11 software. The phylogenetic trees were rooted at the midpoint. The numbers at the nodes indicate the maximum likelihood bootstrap values for 1000 repeats within the specified model.

**Figure 13:**
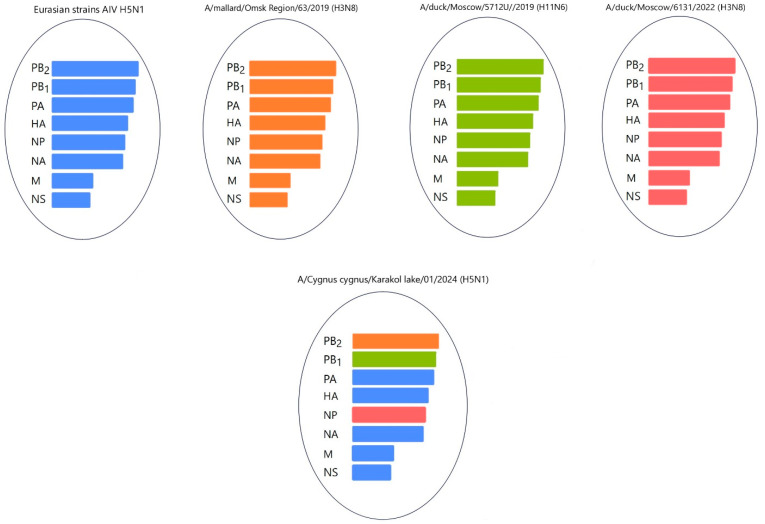
Hypothetical reassortment events of the A/*Cygnus cygnus*/Karakol lake/01/2024(H5N1) viruses. The eight genes are shown in Table 1 and are as follows: PB2, PB1, PA, HA, NP, NA, M, and NS. The colors of the bars indicate the different sources of the gene segments.

**Figure 14:**
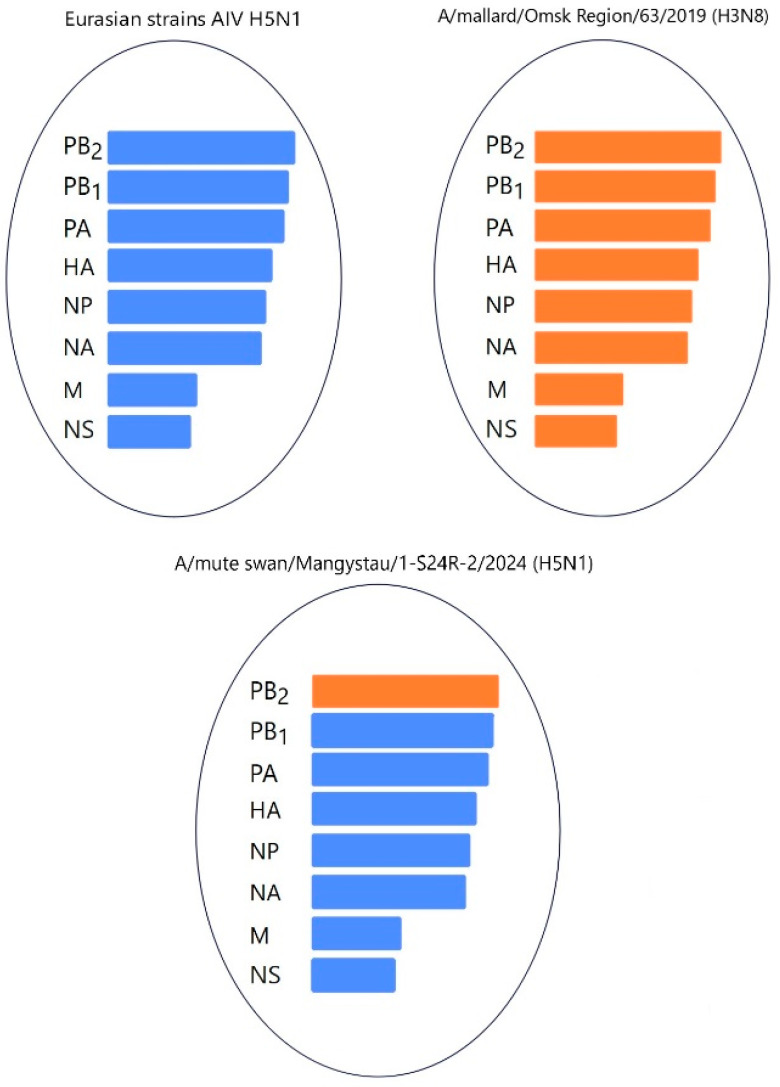
Hypothetical reassortment events of the A/*mute swan*/Mangystau/1-S24R-2/2024(H5N1) viruses.

Gene PB2 of the three strains (A/*mute swan*/Mangystau/1-S24R-2/2024(H5N1), A/*Cygnus cygnus*/Karakol lake/01/2024(H5N1)) obtained in this study clustered with low-pathogenic avian influenza viruses (LPAIVs) and had the highest nucleotide identity (from 99, 53% to 99.75%) with the H3N8 virus isolated from ducks (mallards) in the Omsk region of Russia in 2019 (Figure 13 and Figure 14, Table S3).

**Figure 15:**
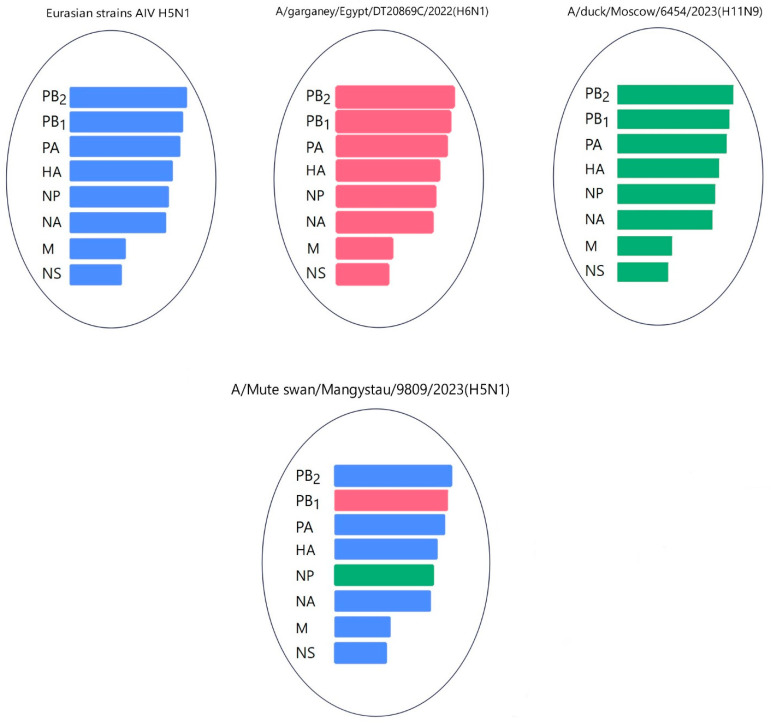
Hypothetical reassortment events of the A/*Mute swan*/Mangystau/9809/2023(H5N1) viruses.

Gene PB1 of strains A/*Cygnus cygnus*/Karakol lake/01/2024(H5N1)) was identical to the AIV H11N6 isolated from ducks in the Moscow region in 2019 (Figure 13 and Table S3). The sequence of the PB1 gene of strain A/*Mute swan*/Mangystau/9809/2023(H5N1) was closest (96.59% identity) to the AIV A/H6N1 isolated from ducks (garganeys) in Egypt in 2022 (Figure 15 and Table S3).

The nucleotide sequence of the NP gene of strains A/*Cygnus cygnus*/Karakol lake/01/2024(H5N1) had 98.91% identity with strain A/duck/Moscow/6131/2022 (A/H3N8) (EPI_ISL_19230715) (Figure 13, Table S3). The NP gene of strain A/*Mute swan*/Mangystau/9809/2023(H5N1) was close (99.19% identity) to strain A/duck/Moscow/6454/2023 (A/H11N9) (Figure 15, Table S3).

These data indicate that the PB2, PB1, and NP of Kazakhstani strains may be derived from low-pathogenic avian influenza viruses (LPAIVs), indicating that the H5N1 viruses in this study are novel reassortants.

## 4. Discussion

In the winter of 2023/2024, mass swan mortality was recorded on Lake Karakol. During the period from 21 December 2023 to 25 January 2024, 1132 dead swans were found on the shores of Lake Karakol. As a result of our research, it was determined that the death of the birds occurred as a result of an outbreak of avian influenza type A/H5N1.

The swan mortality in the winter of 2023/2024 is the largest recorded mortality of this species in the region. The proportion of the epizootic in the winter of 2023/2024 was probably influenced by the high concentration of migratory birds at Lake Karakol. Previously, the largest outbreak of avian influenza in the Caspian Sea region, with mass mortality of 400 mute swans, was registered in 2005 [18].

The observation of migratory birds at Lake Karakol during the epizootic showed that due to the sharp cold weather, all migrating birds did not stay long at this place and, after a short stay, flew away from the lake. The epizootic on Lake Karakol lasted more than 1 month, and during this time, there were a lot of sick and dead birds on the lake, which released the virus into the environment. As is known, the influenza virus can persist in water for up to several days, and most migratory birds—including swans that stayed at this lake—could have been infected with the avian influenza virus and carried this virus to other regions.

In this study, three strains of influenza A/H5N1 2.3.4.4.4.b virus were isolated from swans at Lake Karakol on the eastern coast of the Caspian Sea, which is a key stopover site for swans during their migration. The results of sequencing and further phylogenetic analysis showed that the strains had a high degree of homology (>98.0%) in major genes (H, N, NS, NP, and M) with epidemic strains previously isolated from different regions of the Eurasian continent. The close phylogenetic relationships of some isolated viruses with Eurasian viruses suggest broad links and transmission routes throughout Eurasia.

Phylogenetic analysis of the viruses isolated during the epizootic showed that three new H5N1 viruses can be classified as reassortants that have different combinations of PB2, PB1, and NP from low-pathogenic avian influenza viruses (LPAIVs). The findings suggest that different reassortant avian influenza viruses circulated during different periods of the epizootic. At the beginning of the epizootic (December 2023), a virus with two reassortments in the PB1 and NP genes was circulating. In early January 2024, the A/*Cygnus cygnus*/Karakol lake/01/2024(H5N1) and A/*Mute Swan*/Karakol lake/02/2024(H5N1) viruses with reassortments in the PB2, PB1, and NP genes (isolated by RIBSP) and the A/*mute swan*/Mangystau/1-S24R-2/2024(H5N1) virus with one reassortment in the PB2 gene (isolated by IMV together with KazNARU) were circulating.

Amino acid analysis showed that all isolated viruses had a multi-base amino acid cleavage site (PLREKRRRKR/G) of the HA protein and were highly pathogenic [28]. The N110S/T139P/T156A mutations in the HA protein indicate that these viruses may have strong binding activity to α-2,6-linked SA receptors, which may increase their ability to be transmitted to mammals (Table S4) [29]. For the PB2, PB1, and NS1 genes, in all isolates, we found several amino acid sites that are associated with increased polymerase activity and increased pathogenicity in mammals (Table S4) [30,31,32,33,34]. Moreover, several molecular markers that may cause increased virulence in mammals were found in PB2, NP, and NS1 (Table S4) [30,35].

Thus, the genetic analysis shows that the virus that caused the death of swans at Lake Karakol in the winter of 2023/2024 is genetically different from the strains that caused previous outbreaks among domestic and wild birds in Kazakhstan, which excludes the possibility of the introduction of HPAIV from other regions of Kazakhstan. Given the migration route mentioned above, it can be assumed that HPAIV was introduced in December by migrating birds from other countries. Although swans are considered to be one of the most susceptible species to the H5N1 avian influenza virus, it is unlikely that birds that permanently reside on this lake retained the virus in the flock. The probability that the swans were infected by poultry is also low, as there were only 404,680 poultry kept in various types of farms in the Mangystau region as of 5 January 2024, and there have been no reports of diseased poultry with influenza virus on these farms.

We previously showed that the wetlands of Kazakhstan play an important role in central Asia as a center of avian influenza virus transmission, linking East Asian migration routes with European migration routes and vice versa [36]. Our results confirmed that wild birds (including swans) can carry the influenza virus during migration, resulting in long-distance transmission. The results provide important evidence of virus transmission along the migration route. This study showed that isolated H5N1 viruses underwent complex reassortment during long-distance spread along different avian migration routes. We found amino acid substitutions in PB2, PB1, NP, and NS1 segments that are critical for increased virulence or adaptation to mammals. All these factors require further study of avian influenza viruses on major migration routes and nesting and wintering sites. The Veterinary Service of Kazakhstan will be advised to organize regular monitoring to strengthen surveillance of the avian influenza virus among wild birds. This will help avoid potential threats to poultry and human health caused by the highly pathogenic A/H5N1 virus.

## 5. Conclusions

The results of this research showed that the disease and death of birds at Lake Karakol were caused by the highly pathogenic avian influenza A/H5N1 virus of genetic line 2.3.4.4.b. A phylogenetic analysis of the three isolated viruses showed close genetic relatedness in major genes (H, N, NS, NP, and M) to other common AIV isolates previously found in Eurasia. The close phylogenetic relationships of some of the isolated viruses with Eurasian viruses suggest wide connections and routes of virus transmission throughout Eurasia. Studies have shown that the isolated H5N1 viruses have undergone complex reassortment during long-distance spread along various bird migration routes. These viruses are new reassortants, since they had PB2, PB1, and NP from low-pathogenic avian influenza viruses (LPAIVs). In addition, we found amino acid substitutions in the PB2, PB1, NP, and NS1 segments that are critical for enhancing virulence or adaptation to mammals. All these factors require further study of avian influenza viruses in the main migration routes, nesting sites, and wintering areas. It is recommended that the Veterinary Service of Kazakhstan organize regular monitoring to strengthen epidemiological surveillance for avian influenza virus in wild birds. This will help to avoid potential threats to the health of poultry and humans caused by the highly pathogenic A/H5N1 virus.

## Figures and Tables

**Table 1:** Characteristics of A/H5N1 virus isolates.

#	Strain Name	Sampling Date	Originating Lab	Bird Species	Genbank (Access Numbers)
1	A/*Mute swan*/Mangystau/9809/2023(H5N1)	26 December 2023	IMV	*Mute swan*	PQ151799.1 PB2 PQ151800.1 PB1 PQ151801.1 PA PP346196.1 HA PQ151802.1 NP PP346197.1 NA PP346198.1 M PP346199.1 NS
2	A/*Cygnus cygnus*/Karakol lake/01/2024(H5N1)	10 January 2024	RIBSP	*Whooper swan*	P348302 PB2 PP348303 PB1 PP348304 PA PP348305 HA PP348306 NP PP348307 NA PP348308 M PP348309 NS
3	A/*Mute swan*/Karakol lake/02/2024(H5N1)	10 January 2024	RIBSP	*Mute swan*	PP989359 PB2 PP989360 PB1 PP989361 PA PP989362 HA PP989363 NP PP989364 NA PP989365 M PP989366 NS

## Data Availability

The consensus sequences of the viruses analyzed in this study were submitted to the NCBI and GISAID EpiFlu™ databases under the accession numbers reported in Table 1.

## Supplementary Materials

The following supporting information can be downloaded at https://www.mdpi.com/article/10.3390/ani14223211/s1.
