# Supplementary material for: Reassortants of the Highly Pathogenic Influenza Virus A/H5N1 Causing Mass Swan Mortality in Kazakhstan from 2023 to 2024
Source: Animals (Basel). 2024 Nov 8;14(22):3211. doi: 10.3390/ani14223211 (PMC11591535; doi:10.3390/ani14223211)
Supplement: Supplementary file 1 [file animals-14-03211-s001.zip › Table S1.pdf]

Supplementary material

Table S1. List of primers and probes for RT-PCR and rRT-PCR used in this study.

Name	Sequence	Product size, bp	References
Avian influenza A	rRT-PCR (M+25) AGATGAGTCTTCTAACCGAGGTCG	99	[23]
	rRT-PCR (M-124) TGCAAAAACATCTTCAAGTYTCTG		
	rRT-PCR probe (M+64) FAM-TCAGGCCCCCTCAAAGCCGA-TAMRA		
Primers used for subtyping of HA genes of AI viruses			
H1	H1-883F YDTCGATGCTCCRGTYCAY	391	[22]
	H1-1273R TGYTCYTTRCCYACYGCWGTG		
H3	H3-919F GYATYACTCCWAATGGAAGC	376	[22]
	H3-1294R ATTCTYCCTTCYACTTCDGA		
H4	H4-758F TGGACWATTGTRGADCCWGGA	421	[22]
	H4-1179R GCTGCCTGRGTVGAYTTGAG		
H5	H5-918F CCARTRGGKGCKATAAAYTC	249	[22]
	H5-1166R KGTCTGCWGCRTAYCCRCTY		
H7	H7-937F ATYAAYMSYAGRRCWGTRGG	241	[22]
	H7-1177R GATCWATTGCHGAYTGRGTG		
Primers for subtyping NA genes of AI viruses			
N1	N1-54F TCARTCTGYATGRYAAYTGG	245	[24]
	N1-298R GGRCARAGAGAKGAATTGCC		
N2	N2-59F TYTCTMTAACYATTGCRWCARTATG	278	[24]
	N2-336R GARTT GTCYT TRGAR AAVGG		
N6	N6-57F AGGAATGACACTATCSGTAGTAAG	264	[24]
	N6-307R GAYAGRATRTGCCATGAGTTYAC		
N7	N7-53F TCWGGAGTGGCMATAGCACT	261	[24]
	N7-313R CACKACCCAYCCTTCAACWTTG		
N8	N8-93F CATRTVGTBAGYATYAYARTAAC	137	[24]
	N8-209R ACAYTRGYATTGTRCCATTG		
N9	N9-64F GTAATAGGCACRATYGCAGT	227	[24]
	N9-290R CCTTTRGTYARRTTATTGAA		
Sequencing primers			
PB2	Ba- PB2-1: TATTGGTCTCAGGGAGCGAAAGCAGGTC	2341+29	[25]
	Ba- PB2-2341R: ATATGGTCTCGTATTAGTAGAAACAAGGTCGTT T		
PB1	Ba- PB1-1: TATTGGTCTCAGGGAGCGAAAGCAGGCA	2341+29	[25]
	Ba- PB1-2341R: ATATGGTCTCGTATTAGTAGAAACAAGGCATTT		
PA	Ba- PA-1: TATTGGTCTCAGGGAGCGAAAGCAGGTAC	2233+29	[25]
	Ba- PA-2233R: ATATGGTCTCGTATTAGTAGAAACAAGGTACTT		
HA	Ba- HA-1:	1778+29	[25]

	TATTGGTCTCAGGGAGCGAAAGCAGGGG		
	Ba- NS-890R: ATATGGTCTCGTATTAGTAGAAACAAGGGTGTTTT		
NP	Ba- NP-1: TATTGGTCTCAGGGAGCGAAAGCAGGGTA	1565+29	[25]
	Ba- NP-1565R: ATATGGTCTCGTATTAGTAGAAACAAGGGTATTTTT		
NA	Ba- NA-1: TATTGGTCTCAGGGAGCGAAAGCAGGAGT	1413+29	[25]
	Ba- NA-1565R: ATATGGTCTCGTATTAGTAGAAACAAGGAGTTTTTT		
M	Ba- M-1: TATTGGTCTCAGGGAGCGAAAGCAGGTAG	1027+29	[25]
	Ba- M-1027R: ATATGGTCTCGTATTAGTAGAAACAAGGTAGTTTTT		
NS	Ba- NS-1: TATTGGTCTCAGGGAGCGAAAGCAGGGTG	890+29	[25]
	Ba- NS-890R: ATATGGTCTCGTATTAGTAGAAACAAGGGTGTTTT		
F: forward of the primer, R: reverse of the primer			

22. Tsukamoto, K.; Ashizawa, T.; Nakanishi, K.; Kaji, N.; Suzuki, K.; Okamatsu, M.; Yamaguchi, Sh.: Mase, M. All Rights Reserved. Subtyping of Avian Influenza Viruses H1 to H15 on the Basis of Hemagglutinin Genes by PCR Assay and Molecular Determination of Pathogenic Potential. *Journal of clinical microbiology.* **2008**, 46(9), 3048–3055

23. Spackman, E.; Senne, D.A.; Myers, T.J.; Bulaga, L.L.; Garber, L.P.; Perdue, M.L.; et al. Development of a real-time 630 reverse transcriptase PCR assay for type A influenza virus and the 631 avian H5 and H7 hemagglutinin subtypes. *J Clin Microbiol.* **2002**, 40: 632, 3256–3260.

24. Tsukamoto, K.; Ashizawa, T.; Nakanishi, K. Use of Reverse Transcriptase PCR To Subtype N1 to N9 Neuraminidase Genes of Avian Influenza Viruses. *J Clin Microbiol.* **2009,** 47(7): 2301–2303.

25. Hoffmann, E.; Stech, J.; Guan, Y.; Webster, R. G.; Perez, D. R. Universal primer set for the full-length amplification of all influenza A viruses. *Arch Virol.* **2001,** 146: 2275–2289.
