# Supplementary material for: Reassortants of the Highly Pathogenic Influenza Virus A/H5N1 Causing Mass Swan Mortality in Kazakhstan from 2023 to 2024
Source: Animals (Basel). 2024 Nov 8;14(22):3211. doi: 10.3390/ani14223211 (PMC11591535; doi:10.3390/ani14223211)
Supplement: Supplementary file 1 [file animals-14-03211-s001.zip › Table S2.pdf]

Table S2 - Accession numbers used in this study

PB2	MW873259, EPI ISL 9572802, MW934689, MW872767, EPI ISL 503357, EPI ISL 11633709, EPI ISL 11633616, EPI ISL 11633613, MW3864, EPI ISL 1096149, LC121425, ON682729, MT020275, EPI ISL 503359, EPI ISL 503358, ON505898, EPI ISL 400267, EPI ISL 400275, PP267962, PP989359, PP348302
PB1	EPI ISL 403713, EPI ISL 403705, OK559469, MN208036, LC121418, MN006986, EPI ISL 7595028, EPI ISL 18537218, MW188635, EPI ISL 697696, EPI ISL 403726, EPI ISL 337411, EPI ISL 1063993, EPI ISL 2234807, EPI ISL 14390387, EPI ISL 697685, EPI ISL 18647455, EPI ISL 18526633, EPI ISL 13243697, EPI ISL 943555, EPI ISL 7987334, EPI ISL 16997727, PP989360, PP348303, EPI ISL 14931848, EPI ISL 18916729, EPI ISL 190140, PP267963, EPI ISL 18956165
PA	EPI ISL 18787952, EPI ISL 18787982, EPI ISL 18787976, EPI ISL 18912911, EPI ISL 18886288, PP267964, PP348304, PP989361, EPI ISL 18591993, EPI ISL 18591990, OR783399, EPI ISL 17512128, EPI ISL 17246921, EPI ISL 17716087, OP590396, OR634758, OP874614, KY635563, ON682727, MT020269, MW188636, MT439902
HA	EPI ISL 18612235, EPI ISL 18612186, EPI ISL 18612185, EPI ISL 18513244, EPI ISL 18612269, EPI ISL 18612234, EPI ISL 18787976, EPI ISL 18787982, EPI ISL 18854215, EPI ISL 18854057, PP267965, EPI ISL 18934797, PP989362, PP348305, OR783400, OQ711838, OP597612, PP150344, PP191107, OP590397, OP597636, OP491851, OQ804411, OQ632819, OQ632820
NP	PP989363, PP348306, EPI ISL 16633751, EPI ISL 18634820, EPI ISL 7975818, OR664651, EPI ISL 502593, MT406812, EPI ISL 14931849, EPI ISL 14931848, OQ794104, EPI ISL 5463806, EPI ISL 5463804, EPI ISL 5463805, EPI ISL 4056296, MN208011, MW934977, KX979542, OQ794050, MW872761, OR783415, PP267966, OR783401
NA	PP989364, PP348307, EPI ISL 18934797, PP267967, EPI ISL 18812084, EPI ISL 18540525, EPI ISL 18591987, EPI ISL 18591988, EPI ISL 18652553, EPI ISL 18652555, EPI ISL 17514129, EPI ISL 17514130, EPI ISL 17514131, EPI ISL 17179653, OP590399, OQ632900, OP874617, OR634761, OP597622, OQ804413, OP597630, PP032041, OL636394, OP030704, OR388765

M	MW275976, MW505352, OP597621, OP597629, PP052966, OR388764, OP030705, OP874618, OP590400, EPI ISL 18652555, EPI ISL 18345257, EPL ISL 18591989, EPL ISL 18591988, EPI ISL 18591987, EPI ISL 18540525, EPI ISL 18934797, PP989365, PP348308, EPI ISL 18867005, PP267968, EPI ISL 18787982, EPI ISL 18787976, EPI ISL 18787952
NS	MT020274, MT020282, EPI ISL 4071629, EPI ISL 337569, MT090307, MW466161, EPI ISL 4071623, OK559484, EPI ISL 18343240, EPI ISL 9012576, EPI ISL 1241001, MT407101, MF693931, EPI ISL 18934797, PP267969, EPI ISL 18787982, EPI ISL 18612180, PP989366, PP348309, EPI ISL 18788874, EPI ISL 18858131, EPI ISL 18683420, EPI ISL 18612231, EPI ISL 18586149, EPI ISL 18591987
