# Supplementary material for: Reassortants of the Highly Pathogenic Influenza Virus A/H5N1 Causing Mass Swan Mortality in Kazakhstan from 2023 to 2024
Source: Animals (Basel). 2024 Nov 8;14(22):3211. doi: 10.3390/ani14223211 (PMC11591535; doi:10.3390/ani14223211)
Supplement: Supplementary file 1 [file animals-14-03211-s001.zip › Table S3.pdf]

Table S3: Sequence identity (%) of A/Cygnus cygnus/Karakol lake/ 01/2024(H5N1) and A/Mute Swan/Karakol lake/02/2024(H5N1) strains compared to sequences from GenBank and GISAID database.

Isolate	Gene	Strain	ID	Collection date	Sequence Identity %
A/mute swan/Mangystau/ 1-S24R-2/2024 (H5N1) KazNARU+IMV	PB2	A/mallard/Omsk Region/63/2019 (A/H3N8)	EPI_ISL_400275	31-Aug-2019	98.75
	PB1	A/mute-swan/Croatia/85-1_24VIR670-4/2023 (A/H5N1)	EPI_ISL_18956166	19-Dec-2023	99.74
	PA	A/chicken/Austria/24013031-002/2024 (A/H5N1)	EPI_ISL_18916771	01-Feb-2024	99.42
	HA	A/Eurasian teal /Israel/5/2023 (A/H5N1)	EPI_ISL_19331432	28-Dec-2023	99.77
	NP	A/duck/Austria/24014885-014/2024 (A/H5N1)	EPI_ISL_18983110	02-Feb-2024	99.68
	NA	A/cygnus-olor/Romania/17019_24VIR1002-4/2023 (A/H5N1)	EPI_ISL_18956174	28-Dec-2023	99.31
	M	A/duck/Austria/24014885-014/2024 (A/H5N1)	EPI_ISL_18983110	02-Feb-2024	99.70
	NS	A/mute-swan/Croatia/85-1_24VIR670-4/2023 (A/H5N1)	EPI_ISL_18956166	19-Dec-2023	99.77
A/Cygnus cygnus/Karakol lake/ 01/2024(H5N1), A/Mute Swan/Karakol lake/02/2024(H5N1)	PB2	A/mallard/Omsk Region/63/2019 (A/H3N8)	EPI_ISL_400275	31-Aug-2019	98.54
	PB1	A/duck/Moscow/5712U/2019 (A/H11N6)	EPI_ISL_697685	21-Oct-2019	98.58
	PA	A/chicken/Austria/24013031-002/2024 (A/H5N1)	EPI_ISL_18916771	01-Feb-2024	99.46
	HA	A/Eurasian teal /Israel/5/2023 (A/H5N1)	EPI_ISL_19331432	28-Dec-2023	99.23
	NP	A/duck/Moscow/6131/2022 (A/H3N8)	EPI_ISL_19230715	17-Oct-2022	98.91
		A/duck/Moscow/6135/2022 (A/H6N2)	EPI_ISL_19230712	08-Oct-2022	98.78
	NA	A/swan/Moldova/9045_24VIR442-11/2023 (A/H5N1)	EPI_ISL_18913195	21-Dec-2023	99.16
	M	A/peregrine-falcon/Cyprus/24VIR1053/2024 (A/H5N1)	EPI_ISL_18956184	05-Jan-2024	100%
	NS	A/Eurasian teal /Israel/5/2023 (A/H5N1)	EPI_ISL_19331432	28-Dec-2023	99.18
A/Mute swan/Mangystau/ KZ/9809/2023	PB2	A/duck/Saratov/29-08V/2021 (A/H5N1)	EPI_ISL_5463802	30-Sep-2021	98.03
	PB1	A/garganey/Egypt/DT20869C/2022(H6N1)	OR786178.1	21-Dec-2022	96.59
	PA	A/chicken/Italy/23VIR10336-32/2023 (A/H5N1)	EPI_ISL_18612235	16-Nov-2023	99.21

	HA	A/peregrine-falcon/Cyprus/24VIR1053/2024 (A/H5N1)	EPI_ISL_18956184	05-Jan-2024	99.37
	NP	A/duck/Moscow/6454/2023 (A/H11N9)	EPI_ISL_19230711	16-Nov-2023	99.19
	NA	A/cygnus-olor/Romania/17019_24VIR1002-4/2023 (A/H5N1)	EPI_ISL_18956174	28-Dec-2023	99.29
	M	A/domestic_duck/Czech_Republic/2268-4/2024 (A/H5N1)	EPI_ISL_19338431	04-Feb-2024	98.74
	NS	A/mute-swan/Croatia/85-1_24VIR670-4/2023 (A/H5N1)	EPI_ISL_18956166	19-Feb-2024	99.44
