# Supplementary material for: Reassortants of the Highly Pathogenic Influenza Virus A/H5N1 Causing Mass Swan Mortality in Kazakhstan from 2023 to 2024
Source: Animals (Basel). 2024 Nov 8;14(22):3211. doi: 10.3390/ani14223211 (PMC11591535; doi:10.3390/ani14223211)
Supplement: Supplementary file 1 [file animals-14-03211-s001.zip › Table S4.pdf]

Supplementary material

Table S4: Amino acid substitutions associated with mammalian adaptations.

Gene	Mutation			Associated effect
	A/swan/Kazakhstan/ RPCMV-9809/2023 (H5N1)	A/mute swan/ Mangystau/1-S24R-2/2024(H5N1)	A/Cygnus cygnus/ Karakol lake/ 01/2024(H5N1)	
PB2	L89V	L89V	L89V	Increased virulence in mice [31]
	G309D, T339K, R477G, I495V, A676T	G309D, T339K, R477G, I495V, A676T	G309D, T339K, R477G, I495V, A676T	Compensates for 627K deficiency. Interspecies transmission of influenza A virus [31]
	627E	627E	627E	The PB2 627E mutation is involved in the adaptation of H5N1 viruses in birds [32]
	K482R	K482R	K482R	Increased polymerase activity in mammalian cell line [31]
	A588V	A588V	A588V	A Increased virulence in mice [30]
	Q591K	Q591K	Q591K	Q Increased virulence in mice [30]
	V598T	V598T	V598T	T Increased virulence in mice [30]
	D701N	D701N	D701N	D Increased virulence in mice [30]
PB1	13P	L13P	L13P	Participates in the adaptation of H5N1 viruses to mammalian hosts [33, 34]
HA	PLREKRRKR/GLF	PLREKRRKR/GLF	PLREKRRKR/GLF	Amino acid composition of the HA cleavage site [29]
	N110S	N110S	N110S	S Increased binding affinity to α-2,6 sialic acid receptors [30]
	T139P	T139P	T139P	P Increased binding affinity to α-2,6 sialic acid receptors [30]
	T156A	T156A	T156A	A Increased binding affinity to

				α-2,6 sialic acid receptors [30]
NP	F313Y	F313Y	-	F Increased viral replication [30]
NS1	-	ESEV	ESEV	The four C-terminal residues of the ESEV of the NS1 protein are the XS/TXV-type PDZ ligand domain, and represent a virulence determinant. [36]
	-	149A	149A	Determines the ability of H5N1 avian influenza virus to counteract the production of interferon alpha (IFN-α) and interferon beta (IFN-β) in host cells [35]
	-	P42S	P42S	S Increased virulence in mice [29]

29. Luczo, J.M.; Stambas, J.; Durr, P.A.; Michalski, W.P.; Bingham, J. Molecular pathogenesis of H5 highly pathogenic avian infuenza: the role of the haemagglutinin cleavage site motif. *Rev Med Virol*, **2015**; 25(6): 406–430.
30. Cho, A.Y.; Si, Y-J.;, Lee, D-Y.; Kim, D-J.; Kim, D.; Jeong, H.; Song, C-S.; Lee, D-H.; Index case of H5N1 clade 2.3.4.4b highly pathogenic avian influenza virus in wild birds, South Korea, November 2023. *Front. Vet. Sci*. **2024**, 11:1366082.
31. Li, J.; Ishaq, M.; Prudence, M.; Xi, X.; Hu, T.; Liu, Q.; Guo, D. Single mutation at the amino acid position 627 of PB2 that leads to increased virulence of an H5N1 avian influenza virus during adaptation in mice can be compensated by multiple muta-tions at other sites of PB2. *Virus Research*. **2009**, 144 1/2), 123–129
32. Horimoto, T.; Kawaoka, Y.; Pandemic threat posed by avian influenza A viruses. *Clin Microbiol Rev*. **2001**, 14(1):129-49.
33. Gabriel G.; Herwig A.; Klenk H-D. Interaction of Polymerase Subunit PB2 and NP with Importin α1 Is a Determinant of Host Range of Influenza A Virus. *Plos Pathogens* **2008**, 8, 4(2).
34. de Jong, M.D.; Simmons, C.P.; Thanh, T.T.; Hien, V.M.; Smith, G.J.; Chau, T.N.; Hoang, D.M.; Chau, N.V.; Khanh, T.H.; Dong, V.C.; Qui, P.T.; Cam, B.V.; Ha do, Q.; Guan, Y.; Peiris, J.S.; Chinh, N.T.; Hien, T.T.; Farrar, J. Fatal outcome of human influenza A (H5N1) is associated with high viral load and hypercytokinemia. *Nature Medicine*. **2006**, 12 (10), 1203–1207.

35. Li Z.; Jiang, Y.; Jiao, P.; Wang, A.; Zhao, F.; Tian, G.; Wang, X.; Yu, K.; Bu, Z.; Chen, H. The NS1 gene contributes to the virulence of H5N1 avian influenza viruses. *J Virol*. **2006**. 80 : 11115-11123.
36. Jackson, D.; Hossain, Md.J.; Hickman D. A new influenza virus virulence determinant: The NS1 protein four C-terminal residues modulate pathogenicity. *Biological sciences*. **2008**, 105 (11) 4381-4386
